# Exploring the Level of Physical Fitness on Physical Activity and Physical Literacy Among Chinese University Students: A Cross-Sectional Study

**DOI:** 10.3389/fpsyg.2022.833461

**Published:** 2022-03-16

**Authors:** Cheng Zhang, Yong Liu, Shuang Xu, Raymond Kim-Wai Sum, Ruisi Ma, Pu Zhong, Shixiang Liu, Minghui Li

**Affiliations:** ^1^College of Physical Education and Health Sciences, Chongqing Normal University, Chongqing, China; ^2^Children’s Health and Exercise Research Centre (CHERC), Sport and Health Sciences, College of Life and Environmental Sciences, University of Exeter, Exeter, United Kingdom; ^3^School of Physical Education, Chongqing University, Chongqing, China; ^4^Department of Sport Science and Physical Education, The Chinese University of Hong Kong, Hong Kong, Hong Kong SAR, China; ^5^School of Physical Education, Jinan University, Guangzhou, China

**Keywords:** physical literacy, physical fitness, physical activity, university students, cross-sectional study

## Abstract

Physical literacy (PL) has received considerable attention in the field of physical education and physical activity (PA) worldwide. According to recent studies, the level of physical fitness (PF) among Chinese university students is gradually decreasing. This study aims to examine the impact of the PF level (fit/unfit) on PA and PL, as well as the relationships among PF, PA, and PL, in Chinese university students. Participants comprised 798 university students (390 men; mean age, 19.2 ± 1.2 years) in Chongqing, China. Participants completed the tests of vital capacity, cardiorespiratory fitness, muscular strength, and flexibility, according to the National Physical Fitness Measurement Standards Manual (NPFMSM), as well as questionnaires on PA (time spent performing PA at various intensities) and PL. The independent *t*-tests were conducted to examine sex differences in the evaluated variables, and the Pearson’s correlation between each PF test and PL attributes and PA was calculated according to sex. In addition, the independent *t*-tests were conducted to determine whether the PF level had an effect on PL attributes and PA at various intensities. Significant sex differences were found in the PF domains of vital capacity, muscular strength, and aerobic fitness, with higher scores in men than in women (all *p*-values < 0.05), but not in the PF domain of flexibility and total PL score. Furthermore, the PF domains of muscular strength and aerobic fitness were significantly and positively correlated with the PL attributes of confidence and physical competence in both men and women, while the PF domains of vital capacity and aerobic fitness were significantly and positively correlated with the PL attribute of motivation in men. In addition, PL was significantly and positively associated with cardiorespiratory fitness, vital capacity, muscular strength, and flexibility among participants in the fit group. These findings support advocating for increased participation in PA in university students and using PL as a tool to improve PF components.

## Introduction

Physical activity (PA) and physical fitness (PF) are causally related. The WHO recommends that young adults perform at least 60 min of moderate-to-vigorous PA (MVPA) per day, in order to obtain optimal health benefits (Chaput et al., 2020). Despite this, significant declines in PA among college students are prevalent worldwide (Hallal et al., 2012). For example, an astounding 82.5% and 89.8% of 18-year-old male and 21-year-old female Chinese university students, respectively, are physically inactive (i.e., do not meet the WHO guideline for PA) (Chen et al., 2020). In addition, according to a national survey, the prevalence of overweight status and obesity has dramatically increased in Chinese university students (Tian et al., 2016; Jiang et al., 2018). Based on the Report of Nutrition and Chronic Disease Status of Chinese Residents (2020), the body weight of Chinese men and women aged 18–24 years has increased, on average, 3.4 and 1.7 kg, respectively, compared to that in 2015. These facts are alarming, as an insufficient level of PA is closely related to overweight status, chronic disease, mental health problems, and poor social and cognitive health outcomes (Aubert et al., 2018, 2021). Therefore, it is vital to identify the current condition of PA among Chinese university students, identifying corresponding solutions to motivate university students to participate in PA.

In response to this concerning trend, the State Council issued the “National Fitness Program (2016–2020)” to monitor the PF levels in the university level and possibly set a requirement for graduation. The program aims to better meet the growing demand for participation in PA among young people, and clearly illustrates the need for university students to pay more attention to exercise, for a healthier and stronger body (Ng et al., 2014). In brief, the program comprises measurements of the body mass index (BMI), vital capacity, and performance on the sit and reach test, standing long jump, and 800/1,000 m race, with each test score, according to a predesignated criterion. Accordingly, the testing program provides a comprehensive assessment of the body composition, cardiovascular fitness, strength, endurance, and flexibility in university students.

Physical literacy (PL), a multidimensional construct, has been gaining attention in the research community, and scholars have reached a consensus for some of its defining components (Morrow et al., 2013). PL has been defined as “the motivation, confidence, physical competence, knowledge and understanding needed to value and take responsibility for engagement in physical activities for life” (Burgi et al., 2011). Accordingly, movement skills, competence, motivational constructs, and the embodied experience are highlighted as the core attributes of the PL concept (Burgi et al., 2011). PL is holistically conceived, in that its definition includes physical, cognitive, and affective domains. Thus, PL provides an innovative perspective for encouraging movement behaviors, in terms of participation in PA and the development of fundamental movement skills.

Previous studies have examined the relationship between PL and PA to emphasize the importance of PL as a means to encourage an active lifestyle throughout the life course (Niederer et al., 2011; Cohen et al., 2014). A recent theoretical exploration proposed an evidence-informed conceptual model linking PL, PA, and health outcomes, including PF, which has provided an important foundational model for empirical studies (Cairney et al., 2019). This previous report proposed reciprocal relationships between PL and PA (Cairney et al., 2019). On the one hand, PL is important for sustained participation in PA; on the other hand, the development of PL is enriched by both unstructured (e.g., free play and recreational pursuits) and structured PA (e.g., sport and physical education) (Cairney et al., 2019). The multidimensional structure of PL is considered important in maintaining lifelong participation in PA and the development of PF (Kirschenbaum et al., 1985). For example, individuals with a proficiency in fundamental movement skills are more likely to have the motivation and confidence supporting participation in PA, which leads to the development of PL later in life (Kirschenbaum et al., 1985).

The PL domains (i.e., physical competence, motivation and confidence, and knowledge) are intercorrelated with each other and are essential for supporting participation in PA during adolescence and adulthood (Peckham et al., 1983). The attributes of physical competence in the multidimensional construct of PL have close connections with the domains of PF, and as such, the two constructs are connected through common underpinning components. A previous study have evaluated the impact of a pilot PL-based intervention on PA and PF in university students and have indicated that increasing the PL level may be a promising modality to promote PA among university students (Kwan et al., 2020). In this previous study, 65 university freshmen participated in a 12-week movement skills program, and, with a cluster research design, the core domains of PL, including movement competence, confidence, motivation, and knowledge and understanding, were simultaneously examined (Kwan et al., 2020). The results demonstrated a moderate interaction effect (time by condition) for PA behaviors, as well as for cardiorespiratory fitness (Kwan et al., 2020). The findings from this pilot program suggest that increasing PL may be an effective approach to maintain PF and attenuate the decline in PA behaviors among university students (Kwan et al., 2020). However, because of the insufficient sample size, these findings need to be verified in further studies with larger sample size to provide more substantial evidence. In addition, a highly relevant assumption is that the benefit of increased PL in terms of PA and PF maintenance might differ between those with low and high PF (Kljajević et al., 2022), which can be investigated by using PF test scores to identify fit and unfit groups *via z*-score calculation (Kljajević et al., 2022). Therefore, we aimed to investigate the impact of PF level (fit/unfit) on PL and PA among university students, with a substantial number of participants. Our second aim was to evaluate the relationships among PF, PA, and PL in university students in China.

## Materials and Methods

### Participants and Recruitment

This study adopts a stratified sampling method to recruit participants from four schools located in the Gaoxin district of Chongqing, China, including two universities and two colleges. In total, 798 university students were recruited (390 men, 48.9%), with a mean age of 19.2 years. Data collection was conducted between November 2020 and December 2020. All participants had normal physical health, with no limitations in exercise participation. Informed consent was obtained prior to participation, and the study was approved by the directors of the four selected schools. In addition, ethical approval was obtained by the Institution Review Board of the Ethics Advisory Committee at Chongqing University.

### Measures

#### Physical Fitness

The PF measurements were conducted at the four schools by trained research assistants, according to the National Physical Fitness Measurement Standards Manual (NPFMSM, University version). In total, eight domains of PF were measured, including seven tests from the NPFMSM and one calculated score. All research assistants completed a 2-h training class on the PF measurements to eliminate discrepancies and avoid the introduction of bias, and to unify data collection under consensus. Any questions from the research assistants were resolved.

Participants’ weight (to the nearest 0.1 kg) and height (to the nearest 0.1 cm) were measured. BMI was calculated using the following formula: BMI = weight (kg) divided by the square of height (m). According to WHO criteria, we divided participants into four categories, namely, low weight (< 18.5 kg/m^2^), normal weight (18.5–23.9 kg/m^2^), overweight (24–27.9 kg/m^2^), and obese (≥ 28 kg/m^2^) (Chen et al., 2020).

Vital capacity was assessed by a spirometer. During the test, the participant held the handle and inhaled as hard as he/she could. Each participant completed the test twice, and the best attempt was recorded.

Aerobic fitness was measured by a long-distance race of 800 m for women and 1,000 m for men. Participants were tested in pairs, starting from a standing position. The score was recorded in minutes and seconds and was validated by two research assistants. The final grade was calculated according to the NPFMSM system, with 100 points as the highest grade.

Muscular strength was assessed *via* the standing long jump, which evaluates the development level of the participant’s lower limb explosive power and physical coordination ability (Chen et al., 2020). The score was determined as the distance from the start line to the heel of the closest foot, and each participant’s best of the three attempts was retained.

Flexibility was evaluated by the sit and reach test. Participants were barefoot and asked to sit on the test instrument, maintaining their legs straight at the start line. Subsequently, they benched their upper body and reached forward as far as possible. The best score of the two attempts was recorded.

#### Physical Activity

PA was evaluated using the International Physical Activity Questionnaire, short form (IPAQ-SF) (Hallal and Victora, 2004). The IPAQ-SF consists of seven items and provides information on the time spent performing vigorous physical activity (VPA), such as aerobics, and moderate physical activity (MPA), such as leisure cycling, walking, and sedentary activities. Participants were asked to recall the number of days (frequency) and the length of time (duration) per day that they performed each activity during the last 7 days or during a normal week. In addition, participants were asked to record the amount of time during which they were sedentary. Data from the questionnaire were summed within each category (i.e., vigorous intensity, moderate intensity, and walking) to estimate the total amount of time spent performing PAs per day. Using the official IPAQ-SF scoring protocol, the total daily PA [metabolic equivalent (MET)⋅min/day] was estimated by summing the product of reported time within each PA category with its corresponding category MET value, expressed as a daily average MET score (Hallal and Victora, 2004).

#### Physical Literacy

The Perceived Physical Literacy Instrument (simplified Chinese version) was adopted to evaluate perceived PL among Chinese university students (Ma et al., 2020). This is an eight-item questionnaire consisting of three domains, namely, confidence and physical competence (e.g., “I possess adequate fundamental movement skills”), motivation (e.g., “I appreciate myself or others doing sports”), and interaction with the environment (e.g., “I have strong communication skills”). Specifically, these three domains, respectively, reflect the need to have strong confidence and physical competence, a positive attitude in doing sports, and to know how to interact with the environment to be physically literate. Each response was rated on a 5-point Likert-type scale ranging from strongly disagree to strongly agree. Adapted from a previous Cantonese version constructed by physical education teachers in Hong Kong, the validity of the current questionnaire was confirmed through confirmatory factor analysis, with factor loadings ranging from 0.60 to 0.92 (Ma et al., 2020).

### Data Collection Procedures

Data collection was conducted at the university campus between November and December 2020. This period was selected because the national PF test was conducted as a requirement for university students at this time. Participants received detailed instructions on how to complete the questionnaires. Following a standardized survey administration protocol, two trained research assistants conducted a survey of PA and PL during regular school time. Participants first completed the PF assessments and then independently completed the survey questionnaires (paper version) in the playing field.

### Data Analysis

Before conducting data analysis, several important assumptions were tested, including, for continuous dependent variables, a lack of outliers, homogeneity of variance (assessed by the Levene’s test), and a normal distribution. Descriptive statistics were conducted to summarize the characteristics of the study sample. The independent *t*-tests were conducted to determine whether there were differences between men and women in the evaluated variables. The Pearson’s correlation between each PF test and PL attributes and PA at various intensities was calculated according to sex. In addition, differences in PL and PA were examined according to PF level as one of the main research questions. Specifically, the total sample was divided into fit and unfit groups based on the *z*-score for the PF tests of vital capacity, cardiorespiratory fitness, muscular strength, and flexibility. Participants were assigned to the unfit group when their *z*-score was < 0, and those with a *z*-score of > 0 were assigned to the fit group. A series of independent *t*-tests were conducted to determine whether the PF level (fit/unfit) had an effect on PL domains and PA at various intensities. Data analyses were conducted using SPSS (version 23; IBM Corp., Armonk, NY, United States). A value of *p* < 0.05 was considered statistically significant.

## Results

### Descriptive Statistics and Sex Differences

Table 1 presents the descriptive characteristics in the study population according to sex. Overall, the significant sex differences were found in the PF tests of vital capacity, muscular strength, aerobic fitness, and sedentary behavior (all *p*-values < 0.05). Men performed better than women on most PF tests, including vital capacity (mean, 4120.4 for men), muscular strength (mean, 229.0 for men), and aerobic fitness (mean, 71.1), but not flexibility, for which women showed slightly higher achievement than men (mean, 17.0). There were no significant sex differences in PL attributes, with similar scores in men and women for these variables. However, significant sex differences were observed for self-perceived PA, with women spending slightly more time walking and performing VPA, while men spent more time performing MPA. In addition, there was a significant sex difference in the time spent sitting, which was greater in women than in men.

**TABLE 1:** Descriptive statistics of the study sample.

Variables	Boys		Girls		Total		*p* *
	N	Mean ± SD	N	Mean ± SD	N	Mean ± SD	
Age	390	18.9 ± 1.0	408	19.5 ± 1.3	798	19.2 ± 1.2	<0.001**
BMI	390	21.4 ± 4.5	408	20.9 ± 3.7	798	21.1 ± 4.1	0.125
**Physical fitness**							
Flexibility (cm)	390	16.8 ± 13.2	408	17.0 ± 6.5	798	16.9 ± 10.3	
Vital capacity (ml)	390	4120.4 ± 943.5	408	3579.4 ± 1092.4	798	3843.8 ± 1056.9	<0.001**
Muscular strength (cm)	390	229.0 ± 24.0	408	199.0 ± 32.1	798	213.7 ± 32.1	<0.001**
Aerobic fitness	390	71.1 ± 15.8	408	67.5 ± 14.5	798	69.3 ± 15.3	0.001*
Overall fitness level	390	0.0 ± 0.7	408	0.0 ± 0.6	798	0.0 ± 0.6	1.000
**Physical literacy**							
Confidence and physical competence	390	11.3 ± 2.2	408	114. ± 2.0	798	11.3 ± 2.1	0.698
Motivation	390	12.1 ± 1.9	408	12.1 ± 1.7	798	12.1 ± 1.8	0.901
Interaction with the environment	390	7.4 ± 1.7	408	7.4 ± 1.6	798	7.4 ± 1.7	0.531
**Physical activity**							
Walking MET-minutes/week	374	2071.6 ± 1683.9	383	2119.6 ± 1964.9	757	2095.9 ± 1830.4	0.719
Moderate MET-minutes/week	304	824.1 ± 1052.6	289	740.8 ± 764.7	593	783.5 ± 923.8	0.273
Vigorous MET-minutes/week	288	1391.5 ± 1391.6	220	1566.8 ± 2420.1	508	1467.4 ± 1906.2	0.305
Sitting time	376	323.0 ± 181.8	388	358.0 ± 179.7	764	340.8 ± 181.5	0.008*

*p < 0.05, **p < 0.01 (two-tailed).

### Relationships Between Physical Fitness, Physical Literacy, and Physical Activity

The associations between PF, PL, and PA are shown in Table 2. The attribute of confidence and physical competence was significantly correlated with muscular strength and aerobic fitness in both men (*r* = 0.11 and *r* = 0.27, respectively) and women (*r* = 0.18 and *r* = 0.15, respectively), while the attributes of motivation and interaction with the environment were significantly associated with aerobic fitness (*r* = 0.13 and *r* = 0.14, respectively) and vital capacity (*r* = 0.11 and *r* = 0.13, respectively) in men. The total level of PL was significantly correlated with vital capacity and aerobic fitness in both men (*r* = 0.11 and *r* = 0.22, respectively) and women (*r* = 0.11 and *r* = 0.11, respectively). MVPA was negatively associated with vital capacity in men (*r* = –0.18) but was positively associated with vial capacity in women (*r* = 0.20). A positive association was also found between muscular strength and MVPA in women (*r* = 0.25). In addition, MVPA was significantly correlated with the attribute of confidence and physical competence in men (*r* = 0.15).

**TABLE 2:** Pearson’s correlation coefficients for physical fitness, physical activity, and physical literacy divided by gender (*N* = 798).

Variable		1	2	3	4	5	6	7	8	9
(1) Flexibility	Male	−								
	Female	−								
(2) Vital capacity	Male	0.09	−							
	Female	−0.26**	−							
(3) Muscular strength	Male	0.08	0.33**	−						
	Female	−0.27**	0.75**	−						
(4) Aerobic fitness	Male	0.07	0.21**	0.19**	−					
	Female	0.15**	0.10*	0.17**	−					
(5) Confidence and physical competence	Male	0.09	0.03	0.11*	0.27**	−				
	Female	−0.01	0.12*	0.18**	0.15**	−				
(6) Motivation	Male	0.04	0.11*	0.03	0.13*	0.46**	−			
	Female	0.10*	0.03	0.05	0.08	0.50**	−			
(7) Interaction with the environment	Male	0.09	0.13*	0.04	0.14**	0.49**	0.51**	−		
	Female	0.09	0.11*	0.08	0.03	0.39**	0.44**	−		
(8) Total PPL	Male	0.09	0.11*	0.08	0.22**	0.83**	0.80**	0.80**	−	
	Female	0.07	0.11*	0.14*	0.11*	0.83**	0.81**	0.74**	−	
(9) MVPA	Male	−0.04	−0.18**	−0.08	−0.06	0.15*	0.05	0.05	0.11	−
	Female	−0.04	0.20**	0.25*	−0.03	0.13	0.02	0.11	0.11	−

*p < 0.05, **p < 0.01 (two-tailed).

**FIGURE 1:**
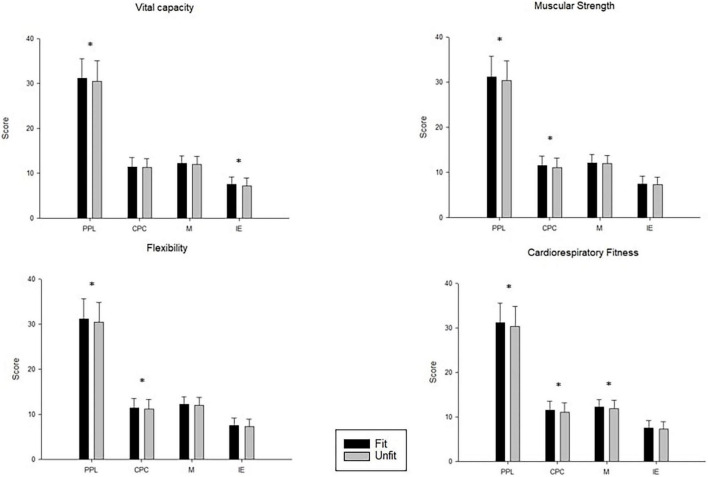
Physical fitness groups (i.e., fit and unfit) and attribute and total physical literacy scores. *p < 0.05.

Figure 1 presents the differences between unfit and fit groups in PL. Participants who were assigned to the fit group for cardiorespiratory fitness, vital capacity, muscular strength, and flexibility had significantly higher total PL scores compared to those in the unfit group. Furthermore, differences between fit and unfit groups in the attributes of confidence and physical competence were also found for all fitness tests, with the exception of the vital capacity test. In contrast, differences between fit and unfit groups in the attribute of motivation only reached significance for the cardiorespiratory fitness test, and the differences between fit and unfit groups in the attribute of interaction with the environment only reached significance for the vital capacity test.

**FIGURE 2:**
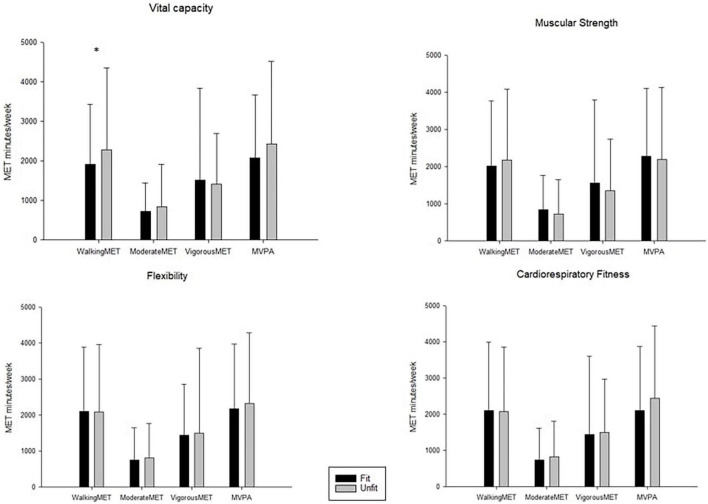
Physical fitness groups (i.e., fit and unfit) and metabolic equivalent of different intensities of physical activity. *p < 0.05.

Figure 2 presents the differences between fit and unfit groups for different PA intensities. A significant difference was observed in the walking MET between the fit and unfit groups for the vital capacity test. However, there were no significant differences between fit and unfit groups for the cardiorespiratory fitness, muscular strength, and flexibility tests in terms of PA participation.

## Discussion

As PA is a predominant contributor to maintaining a healthier life, fostering the development of PL not only promotes lifelong PA but also plays a salient role in maintaining PF (Niederer et al., 2012). It is essential to accurately understand the associations between PF, PA, and PL as theoretically hypothesized by Cairney et al. (2019). This study evaluates the relationships among PF, PA, and PL in Chinese university students. Furthermore, to the best of our knowledge, this is the first study to investigate how the PF level, PA, and PL are interrelated in university students in China.

Significant sex differences were found in the PF tests for vital capacity, muscular strength, and aerobic fitness. In general, men performed better than women on the PF tests, with the exception of flexibility, for which women showed slightly better performance than men. This is consistent with a previous study comprising 2,614 subjects from 11 universities that showed better performance on PF tests in men than in women, which was thought to be due to a more active lifestyle in men, as opposed to a more sedentary lifestyle in women (Sun and Dai, 2006). Furthermore, given the advantages in muscle fiber structure and motor organ in women, it seems reasonable that women have better flexibility than men (Sun and Dai, 2006).

No significant sex differences were found in the PL attributes of confidence and physical competence (men: 11.3 ± 2.2; women: 11.4 ± 2.0), motivation (men: 12.1 ± 1.9; women: 12.1 ± 1.7), and interaction with the environment (men: 7.4 ± 1.7; women: 7.4 ± 1.6). These findings are consistent with those of a recent study, which also did not find any differences between the sex, although their study targeted students aged 12–18 years (Choi et al., 2018). Since their findings were based on a population with a large age variance (6 years), it is plausible that this sex equality may be maintained later in life.

Although PA participation was not statistically associated with PL, the results showed that women spent slightly more time performing both light PA and VPA, while men preferred MPA. These findings are slightly different from those of a previous study, which found that the overall amount of PA was higher in men than in women (Longmuir and Tremblay, 2016). However, our findings do echo a previous study that specifically focused on PA in Chinese university students; according to Yan et al. (2015), men are 1.5 times more likely than women to achieve the minimum PA recommendations for university students. The present findings may differ from previous studies because of the use of the IPAQ-SF questionnaire as a subjective measure for identifying different PA intensities, as Students’ subjective feelings and understanding of PA differ. Therefore, it is essential to interpret the findings with caution. The finding that women spend more time walking than men is consistent with previous studies, showing that women tend to engage in more low-intensity PAs (Zhang, 2019). Furthermore, we found a significant sex difference in the sedentary time, with women spending more time sitting than boys. The sedentary time of university students is mainly spent in class and self-study. The longer accumulation of sedentary time in women may be because women place more value on academic performance and spend more time studying. This is supported by findings from a broad survey in China (Li et al., 2017), which found that women spend more time on academic study than men (7.8 vs. 6.2 h) and generally achieved higher academic performance. In addition, men are generally expected to spend more time participating in sports, which may also contribute to the observed differences between men and women (He et al., 2019). A recent review highlighted that long-term sedentary behavior is deleteriously associated with cardiopulmonary health, indicating that prolonged sedentary time may lead to cardiovascular disease morbidity and mortality (Lavie et al., 2019). In this regard, the present findings are consistent with previous reports that women score lower than men on cardiopulmonary function (vital capacity) and aerobic fitness tests.

The present results are in concordance with previous cross-sectional studies, which have indicated MVPA as significantly associated with muscle strength and vital capacity (Wrotniak et al., 2006). Remarkably, we observed a positive relationship between vital capacity and MVPA irrespective of sex (boys, *r* = 0.18; girls, *r* = 0.2). This finding is consistent with several previous studies. For example, Fuster et al. (2008) studied the relationship between PA and forced vital capacity among a group of young men and women (age, 23.2 ± 1.99) and found that, after adjusting for height, active young people were significantly better than their counterparts in the pulmonary test. Analogously, in a randomized controlled trial conducted by Rocío et al. (2013), both male and female participants showed significantly improved vital capacity after a PA promotion program compared to that in the control group.

In addition, in this study, no significant associations were observed between flexibility and PA and PL. Although flexibility is an indispensable component of PF, the existing literature describes the association between PA and flexibility as “surprisingly limited” (Patnode et al., 2010). A possible explanation for this finding may be that a positive correlation may exist only during a sensitive period. Since our current cross-sectional study targeted a specific age group (e.g., freshman and sophomore), incomplete conclusions may be drawn (Patnode et al., 2010).

In this study, we found that the overall PL score was significantly higher in the fit group than in the unfit group. These findings are consistent with the conclusions of previous studies. For example, in the abovementioned pilot study of the impact of 12-week movement skills program on core domains of PL (Kwan et al., 2020), as various PF indicators improved, the scores on the core domains of PL also improved, with a moderate interaction effect between PF and PL (Kwan et al., 2020). Physical competence is a core domain of PL. Among the attributes of PL, confidence and physical competence showed relatively positive associations with the evaluated aspects of PF. Proficiency in fundamental movement skills and confidence can better promote continuous participation in PA, and long-term PA participation will correspondingly improve PF, including cardiorespiratory fitness and muscular strength (Niederer et al., 2012).

In examining the differences in PA at various intensities according to PF level, we only found a significant difference in the walking MET between fit and unfit groups based on vital capacity. No significance differences in PAs were found between fit and unfit groups for other fitness tests. The walking MET was higher in the unfit group than the fit group. Students with low vital capacity are those who lack physical exercise; their PA time and intensity are relatively low. Accordingly, their low-intensity walking time will be correspondingly higher than that for students with higher vital capacity.

A major strength of this study is that we considered PF in a holistic manner, rather than just focusing on some aspects of PF, to study the relationships between PF, PA, and PL. The overarching PF test evaluates body composition, vital capacity, muscular strength, aerobic fitness, and flexibility. Notably, the results were obtained from a national standard test, which is considered highly reliable. In addition, the sample size of our research was relatively large. Furthermore, participants were selected by stratified sampling. The four universities involved in this study reflected different types of universities in Chongqing, with a total of 798 participants. Furthermore, this study is a pioneer in the exploration of associations among PL, PF, and PA in real-world settings. PL is a comprehensive and influential concept. At present, most of the research on this concept has remained at the theoretical level, and there are few studies on the actual measurement and application of PL.

Despite the abovementioned strengths, our study has the following limitations. First, PF measurement was performed in a school environment, and the test environment was open, hampering the accuracy of the results as compared to that with laboratory tests. As a result, our data should be interpreted with caution. Second, our sample was obtained from four universities in Chongqing, and may not fully represent all university students in China, limiting the universality of our results. Third, the use of questionnaires to collect data regarding PA and PL may involve bias.

## Conclusion

Overall, this study shows a positive relationship between cardiovascular fitness and total perceived PL. Among the attributes of PL, confidence and physical competence showed relatively greater positive associations with the aspects of PF in Chinese university students, while no significant relationship was observed between PF and PA. Our findings support advocating for increased PA participation among university students and using PL as a tool to improve PF. Future studies are needed to target PL as an intervention to improve PA and PL in university students.

## Data Availability Statement

The raw data supporting the conclusions of this article will be made available by the authors, without undue reservation.

## Ethics Statement

The studies involving human participants were reviewed and approved by the Ethics Committee of Chongqing University. The patients/participants provided their written informed consent to participate in this study.

## Author Contributions

CZ, YL, and ML contributed substantially to most of the work, including the study design, statistical analysis, and manuscript write-up. SL supervised and instructed CZ’s work, providing support on the implementation of the study and giving critical suggestions on the study design. YL participated in the study conception and design, data collection, and part of the manuscript write-up, while ML, SX, and RM helped collect and analyze the data, worked with the participants, and reviewed the literature. RS and PZ provided their suggestions on the study design and worked with universities to recruit participants. All authors contributed to the article and approved the submitted version.

## Conflict of Interest

The authors declare that the research was conducted in the absence of any commercial or financial relationships that could be construed as a potential conflict of interest.

## Publisher’s Note

All claims expressed in this article are solely those of the authors and do not necessarily represent those of their affiliated organizations, or those of the publisher, the editors and the reviewers. Any product that may be evaluated in this article, or claim that may be made by its manufacturer, is not guaranteed or endorsed by the publisher.
